# Fast responsive, optical trace level ammonia sensor for environmental monitoring

**DOI:** 10.1186/1752-153X-6-124

**Published:** 2012-10-26

**Authors:** Tobias Abel, Birgit Ungerböck, Ingo Klimant, Torsten Mayr

**Affiliations:** 1 Institute of Analytical Chemistry and Food Chemistry, Graz University of Technology, Stremayrgasse 9, Graz, 8010, Austria

**Keywords:** Optical sensor, Ammonia sensor, Fluorescence sensor, Fish farming, Aqua culture, Dual lifetime referencing, Two wavelength ratiometric referencing

## Abstract

**Background:**

Ammonia is a ubiquitous chemical substance which is created in technical and biological processes and harmful to many different organisms. One specific problem is the toxicity of ammonia in fish at levels of 25 μg/l - a very common issue in today’s aqua culture. In this study we report a development of a fast responsive, optical ammonia sensor for trace concentrations.

**Results:**

Different hydrogels have been investigated as host polymers for a pH based sensing mechanism based on fluorescent dyes. A porous hydrophobic fluoropolymer membrane was used as an ion barrier cover layer to achieve a good ammonia permeability. The sensor’s sensitivity towards ammonia as well as crosssensitivity towards pH-value and salinity, and the temperature dependency have been determined. Two different methods to reference fluorescence signals have been employed to eliminate intensity-based measurement drawbacks.

**Conclusion:**

The presented sensor features high sensitivity and a fast response even at concentrations near 1 ppb. No cross sensitivity towards pH and salinity could be observed and temperature dependency was determined as compensateable. Both referencing approaches prove themselves to be able to provide a simple use of the sensor for in-field applications.

## Background

Ammonia is a very widespread chemical in our world. It is not only present in substances like refrigerants, household cleaners, and (most abundantly) industrial fertilizers, but is also produced in nature by all animal cells [1] resulting from degradation of amino acids [2], food putrefaction [3], excretion, and decomposition of waste and sewage. Due to this broad use and existence of ammonia it can be found in the atmosphere [4], the soil [5], as well as in river- [6] and seawater [7]. Furthermore ammonia is toxic to any kind of animals, from microorganisms [8] to more differentiated life forms [9]. These circumstances lead to an increasing demand for robust, cheap and continuous means of measuring ammonia in environmental monitoring, food processing and medical applications [10]. This study approaches the problem of ammonia monitoring in the aquatic habitat. The developed sensor is suitable for the application in fish farming [11], which itself is an attempt to lessen global overfishing while providing fish for global demands.

Many different methods have been employed for analytical detection of ammonia, such as flow spectrometrics [12], potentiometric electrodes [13], IR absorption [14], amperometric [6] and conductivity [15] measurements, or spectrophotometric approaches based on the Berthelot reaction [16] or on Nessler’s method [12]. However, these methods consume chemicals, need batch separation from the analytical sample, or require sample pretreatment and/ or expensive instrumentation, which prevent these approaches being applied for continuous and simple monitoring tasks. Optical ammonia sensors [17-19] based on pH-indicators can fulfill these conditions, since they can be manufactured for low-cost in high quantities and used with simple instrumentation without sample pretreatment.

In this study we present an optical ammonia sensor based on commercially available materials, simple in both manufacturing and operation, designed to fulfill ammonia monitoring functions as demanded in modern fish farms. The ammonia sensitive layer consists of a hydrogel in which two non-sensitive, fluorescent dyes and a non-fluorescent pH-indicator are entrapped. The two fluorescent dyes and the indicator form a Förster resonance energy transfer [20] (FRET) cascade due to their overlapping emission and absorption spectra of the FRET donor and acceptor respectively. The pH-indicator is protonated in absence and deprotonated in presence of ammonia, changing its absorption spectra and quenching the FRET emission in the presence of ammonia. The analytical information is gained by measuring the cascade’s fluorescence intensity. To overcome problems of varying fluorescence intensities derived from differing layer thicknesses and power attenuation by the instrumentation we employed two different reference methods, one of which even allows ratiometric imaging techniques using the color channels of a CCD camera [21].

## Results and discussion

The general structure of an optical ammonia sensor is based on a hydrophilic polymer, in which the sensing chemistry is immobilized, entrapped sandwich-like between a supporting material and a proton barrier, which permits a permeation of ammonia but prohibits a penetration of protons into the host material.

### Choice of indicators and dyes

The main characteristic which defines the dynamic range of an ammonia sensor based on pH indicators is the pK_a_ of said indicator. Ammonia, which migrates into the host material, deprotonates the indicator and forms an ammonium salt with the indicator as counter ion, as described by equation 1. Though there are some ammonia sensors based on fluorescent indicators, [22-24], the majority rely on absorbance-based pH indicators [17,25-28], in a broader pK_a_ range available.

(1) $${\text{NH}}_{3}+\text{IndH}⇌{{\text{NH}}_{4}}^{+}+{\text{Ind}}^{-}$$

**Figure 1:**
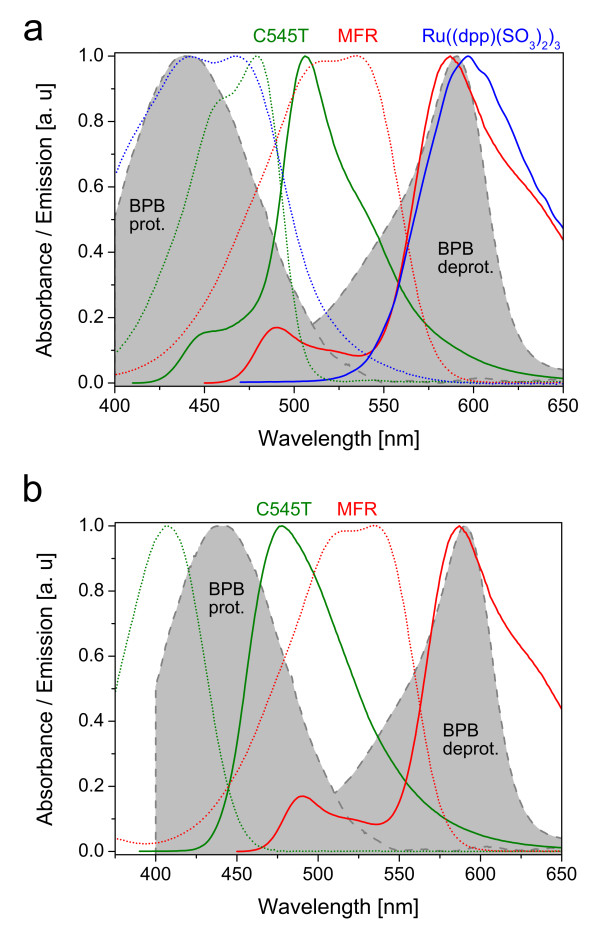
**Absorbance and emission spectra of used sensor compositions.a** shows the DLR system: absorbance (dotted line) and emission (solid line) spectra of Coumarin 545T (green), Macrolex Fluorsecent Red (red) and the Ruthenium complex (blue) as well as the absorbance spectra for the protonated and deprotonated forms of bromophenol blue (grey areas). **b** shows the TWR system: absorbance (dotted line) and emission (solid line) spectra of Coumarin 30 (green), Macrolex Fluorsecent Red (red) as well as the absorbance spectra for the protonated and deprotonated forms of bromophenol blue (grey areas).

The used indicator was bromophenol blue (BPB) with a pK_a_ of 4.1 [17]. The indicator shows different absorbance spectra within the visible field. The deprotonated form shows an absorbance maximum at 600 nm while the protonated form shows an absorbance maximum at 425 nm (see Figure 1 for normalized spectra). Thus, absorbance carries the analytical information. However, absorbance cannot be as easily measured (e. g. by reflection in thin polymer films) as fluorescence. For this reason two more dyes were introduced, which form a FRET cascade: a donor (Coumarin 545T or “C545T”), an acceptor (Macrolex Fluorescent Red G or “MFR”) and a quencher (BPB). The donor is excited and transfers its energy to the acceptor dye, emitting subsequently light at 600 nm. This is prevented, if in presence of ammonia the indicator’s deprotonated form is within the membrane, because the energy will then be passed from MFR onto the indicator and the emission diminishes. This approach offers additional advantages; due to the excitation by energy transfer, photo bleaching of the pH indicator can be minimized. Furthermore, high intensities can be obtained by an increased donor concentration, increasing the absorbance of excitation light [29].

The indicator and dyes necessary for the different referencing techniques are discussed within the appropriate sections.

### Choice of polymers

There are some important properties a polymer has to fulfill to be considered as host material for an ammonia sensor: The host polymer has to stabilize both the hydrophilic ammonium and the hydrophobic protonated indicator, which means that the polymer’s hydrophilicity is a key factor for sensitivity and stability of the sensor. Furthermore the polymer must not have alkaline properties, so that the protonated form of the pH indicator is stable inside the membrane.

**Figure 2:**
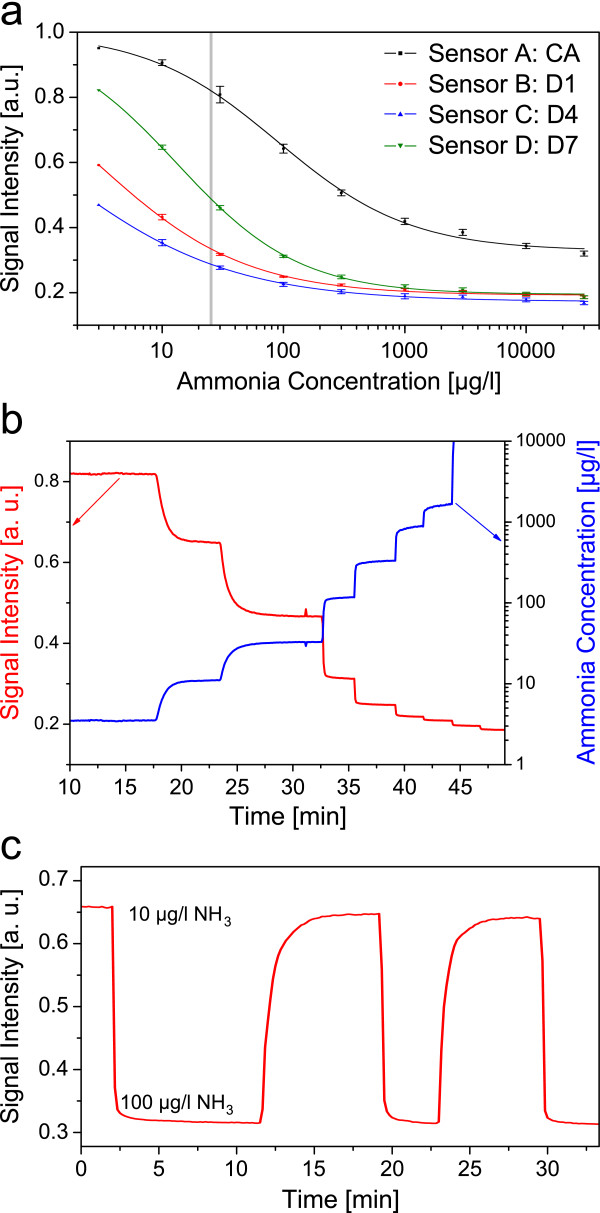
**Sensitivity and response of the ammonia sensor.** (**a**) Calibration plots for ammonia sensors A, B, C, and D in a concentration range between 3 and 30,000 μg/l with a view to the ammonia toxicity level in fish of 25 μg/l (grey line) (mean values from 6 sensor layers); (**b**) time resolved signal response (red graph) and calculated concentrations (blue graph) based on sensor B; (**c**) response and recovery response of sensor B between 10 and 100 μg/l ammonia in 100 mM phosphate buffer at pH 7.2.

Cellulose esters have been used as host polymers [23,24], since they fulfill these requirements. Hydrogels even surpass these characteristics. Their water absorption (70, 50 and 30% for HydroMed D1, D4 and D7 respectively [30] and their strong adhesion properties make them a very suitable choice. As seen in Figure 2a these hydrogels outperform cellulose acetate (CA) in terms of detectable concentration ranges. This is a very crucial requirement, since ammonia displays toxicity towards aquatic and amphibic organisms at concentrations of even 25 μg/l. CA shows 50% of the maximum intensity decrease at about 90 μg/l ammonia, the hydrogels D7, D4 and D1 undermatch this at concentrations of 13, 1 and 2 μg/l respectively. This shows that the sensitivity of the same sensing chemistry is higher in hydrogels than in CA.

### Choice of proton barrier

Ammonia sensors based on a pH sensitive layer require a proton barrier for liquid samples to prohibit a response to the pH of the tested sample. Different materials have been used; silicon being the probably most widespread reported one [23,24,26]. The proton barrier has to feature two properties: impermeability for protons and permeability for ammonia. Facing minimal concentrations during trace measurements, reassuring a high permeability is of the utmost importance to keep the sensors’ response time within reasonable limits.

In the study presented a PTFE-based membrane filter has been used, displaying not only the strong hydrophobic properties of technical fluoropolymers, but also an extraordinary permeability for ammonia [31,32] due to its highly porous structure [33]. Moreover the white, reflective filter surface offers the possibility to keep the analyte sensitive layer thickness below two micro meter whilst maintaining a sufficiently high signal level and minimizing response times. Moreover fluoropolymers offer a high resistivity against biofouling and thus the establishment of biofilms on the sensor’s surface [34], which can lead to deterioration in the sensor performance.

### Sensor response times

Due to the filter membranes used, and the thin polymer layer it was possible to achieve very short response times. The response time t_90_ for a change between 3 μg/l and 10 μg/l is about 120 seconds (see Figure 2b). The t_90_ response time and the recovery response time between 10 μg/l and 100 μg/l were determined to be 60 and 50 seconds respectively (see Figure 2c), decreasing with higher concentrations down to 20 seconds. This sensor clearly outperforms silicon as proton barrier, of which t_90_ response times of 30 [26], 40 [23], even 70 [35] minutes have been reported.

The sensors employing hydrogels (sensor B, C and D) displayed slightly shorter response times between 10 μg/l and 100 μg/l (60, 60 and 50 seconds respectively) than the same setup using cellulose acetate (sensor A, 90 seconds), which demonstrates that the ammonia permeability is in hydrogels higher than in cellulose acetate.

### Cross sensitivity and dependencies

**Figure 3:**
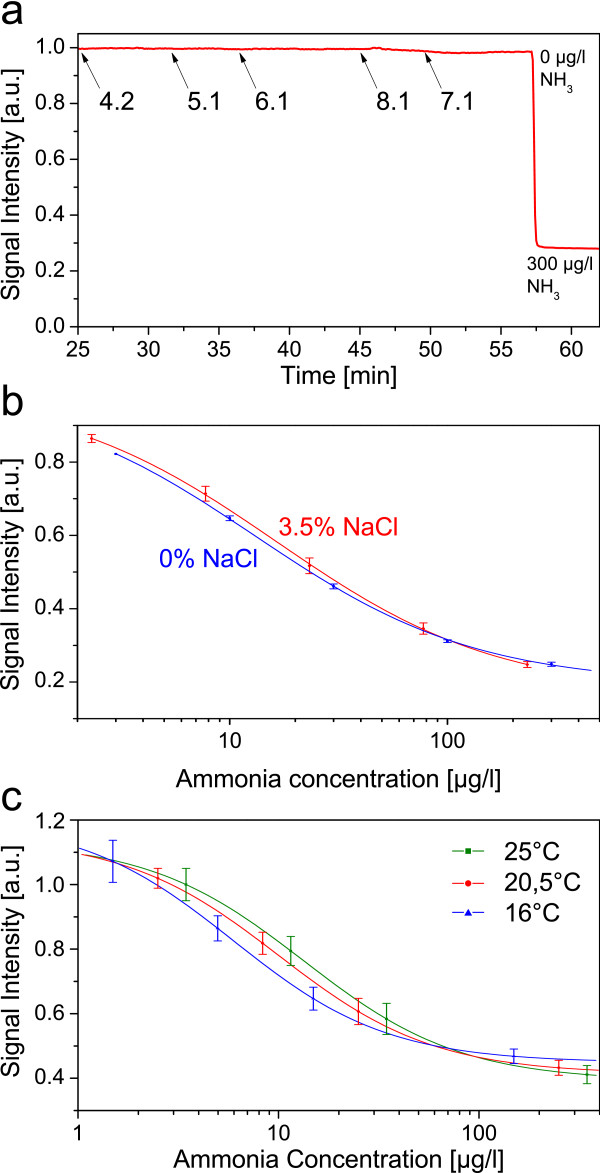
Cross sensitivity of sensor B towards pH (a) and salinity (b, mean value of 4 sensor foils), and temperature dependency (c, mean value of 4 sensor foils).

Different tests were carried out to assess the sensors’ cross sensitivity. The impermeability towards protons is demonstrated as seen in Figure 3a. No change of signal intensity could be registered, even with pH buffers far beyond the pK_a_ value of the used indicator.

Also the sensor’s cross sensitivity towards varying salinities was evaluated (see Figure 3b). Taking into account that the ammonia-ammonium equilibrium changes with salinity (which can be mathematically compensated [36]), the sensor shows virtually no divergence in its calibration in ammonia solutions containing 3.5 %(w/w) sodium chloride compared to sodium chloride free solution.

There is a specific dependency in terms of temperature. Figure 3c show three different calibrations, all carried out with the identical sensor and buffers. The ammonia concentration was corrected for the shifted ammonia-ammonium equilibrium (see experimental section, equation 3). Still, a slight dependency towards temperature was measured within the relevant concentrations. This can be attributed to the universal characteristic of increased fluorescence at lower temperatures and a temperature dependency of the indicator’s pKa. Compensation can be carried out by a simultaneous measurement of the temperature. Dual analyte- temperature sensor where recently published by Borisov et al. [37]. An accurate temperature measurement is mandatory due to the temperature dependency of the ammonia/ammonium equilibrium.

### Dual-Lifetime Referencing (DLR)

All measurements so far discussed are intensity based; those measurements are error-prone in real-world applications. These errors can result from manufacturing inhomogenities, different alignments, or varying performances of read-out units. Therefore, we investigated the application of two different signal referencing methods to overcome the limitations of intensity measurements to obtain a universal and user friendly system suitable for field measurements.

The first reference method employed is Dual Lifetime Referencing [22,38], (DLR). This method requires a phosphorescent reference dye featuring a long decay time, equal excitation and emission wavelengths as the sensing chemistry and no sensitivity towards the analyte or other substances. Silica particles were chosen as host material due to their simple synthesis, their insolubility in organic solvents, and their low oxygen permeability. These particles contained a phosphorescent Ruthenium complex, which would be an oxygen-sensitive probe unless incorporated inside the gas-impermeable spheres. These spheres are evenly dispersed in the sensor cocktail during the manufacturing process and homogenously distributed in the host polymer. Furthermore, it was necessary to decrease the sensors brightness (compared to intensity measurements) by reducing the FRET-pair donor’s concentration to match the luminescence intensities of the sensing chemistry and the reference.

**Figure 4:**
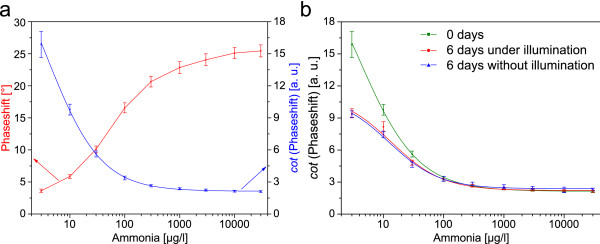
**DLR calibration and longtime stability of sensor E.** (**a**) Calibration of sensor E based on phaseshift measurements (red graph mean value of 2 sensor foils) and calculated correlating cotangent values (blue graph); (**b**) longtime stability comparing illuminated sensors (red graph, mean value of 4 sensor foils) and non-illuminated sensors (blue graph, mean value of 6 sensor foils).

The measurement is carried out with a sinusoidally modulated light source, which excites the indicator and the reference particles. The detected signal exhibits a high or low phase shift, depending on the ratio of the intensities of the reference and the indicator. Figure 4a depicts the calibration curve of the presented DLR-referenced sensor. The red graph shows the measured phase shifts. The blue graph shows the calculated cotangent values, which is directly proportional to the indicators intensity. It has to be remarked, that the high deviation at 3 μg/l is not originated by measurement problems, but is derived from the steepness of the cotangent functions at values next to zero.

Figure 4b shows a long term stability test carried out for 6 days using two different methods: Both sensor batches were stored in a buffer containing 100 μg/l ammonia, and characterized on the first and sixth day of storage. One batch was stored in complete darkness while the other batch was illuminated (6 samples per minutes over the period of 6 days equals 8600 measurement points) to investigate if the drop in signal was caused by photo bleaching. Both tested sensor batches show a drop of the cotangent-values at low ammonia concentrations after 6 days while maintaining the signal values at higher concentration. Since both sensor calibrations drop the same amount it is assumed, that the sensor itself is not prone to photo bleaching but may suffer from leaching or migration problems due to incomplete encapsulation of the indicator and reference dyes. Despite this change in sensor characteristics a recalibration and further use of the tested sensors is still possible. However, for long-time measurements we would recommend an exchange of sensor foils at regular intervals.

### Two Wavelength Ratiometric (TWR)

**Figure 5:**
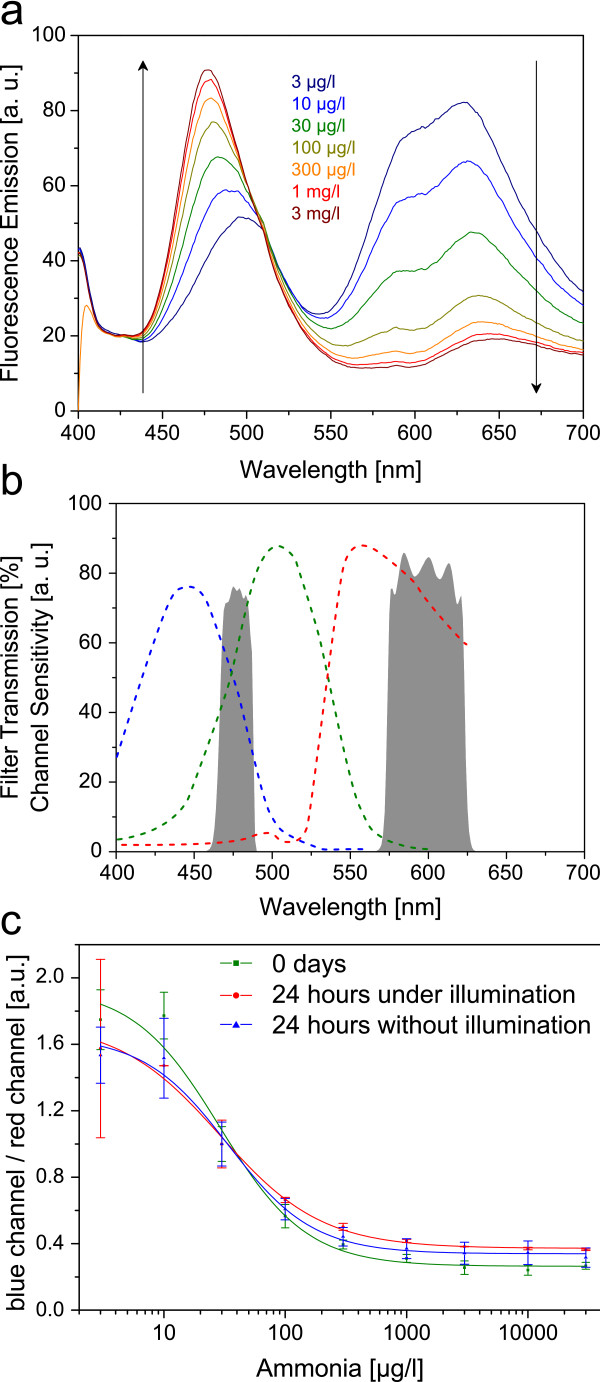
**Sensor and instrumentation characteristics of two-wavelength ratiometric referenced sensor.** (**a**) Fluorescence emission spectra of sensor F at different ammonia concentrations; (**b**) spectral sensitivity for blue, green and red of the color camera’s RGB sensor (dotted lines) and used bandpass filters (grey areas); (**c**) over the period of 24 hours comparing illuminated sensors (red graph mean value of 2 sensor foils) and non-illuminated sensors (blue graph mean value of 4 sensor foils).

A second referencing method was employed; based on ratiometric measurement of two different emission intensities recorded at two different bands of wavelength. The sensing chemistry was modified to gain two different emission peaks with sufficient difference in wavelength and comparable ranges of emission intensity over the whole range of tested ammonia concentrations. The previously used FRET donor, Coumarin 545T, was exchanged for Coumarin 30 (C30), a dye similar in absorbance and quantum yield but featuring shorter absorption and emission wavelengths, which was crucial to obtain two distinctly separated emission peaks. The reduced overlap of the C30 emission and the acceptors absorption spectra (MFR) leads to an decreased FRET efficiency and noticeable emission of the donor in the region of 470 nm (see Figure 5a).

This FRET cascade is combined with the BPB indicator forming the ammonia sensing system. BPB shows two different absorbance bands, its protonated form overlaps the donor’s (C30) emission and the deprotonated form overlaps the acceptor’s (MFR) emission. This leads to an opposing change in intensities with ascending ammonia concentrations, namely an increasing emission of the donor and a decreasing emission of the acceptor (see Figure 5a). The signal can be referenced by dividing the intensity of the 625 nm peak by the intensity of the 470 nm peak, either mathematically from a spectrum (see Figure 5a), by a measurement with two different band pass filters or by imaging techniques with a color camera (see Figure 5b).

For the “two filters approach” a lock-in amplifier was used, connected with a 405 nm LED, a trifurcated fiber bundle and two PMT tubes with two different band pass filters for each emission peak. The emission intensity of both peaks was measured and referenced by dividing the red by the blue channel. Figure 5c depicts a longtime measurement in analogy to Figure 4b. Again a drop in fluorescence intensity can be observed independent from the factor of illumination. Despite this drop the sensor itself is still functional and can be used by further recalibration.

**Figure 6:**
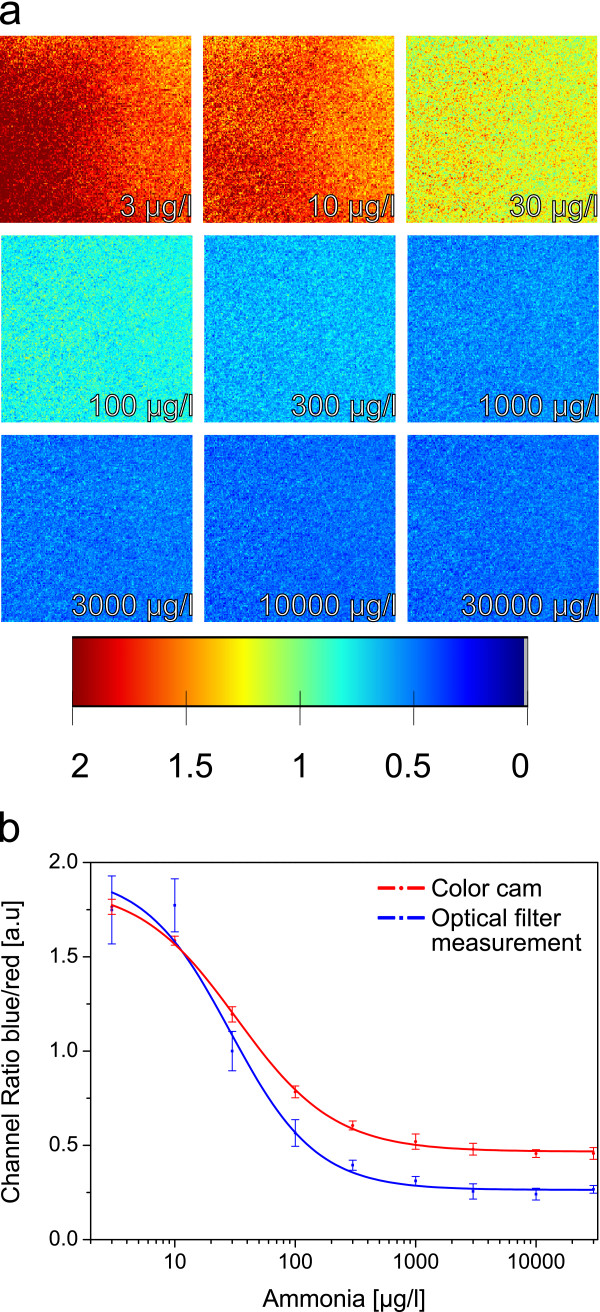
**False color imaging pictures and comparison to optical filter measurement.** False color imaging pictures (**a**) taken with a color camera and processed with MathWorks MATLAB (calculation of blue/red channel ratio) and comparison of the mean values of the channel ratio with a calibration carried out with the optical filter measurement approach.

This ratiometric referencing approach also offers the possibility to use imaging methods [39]. Figure 6 depicts different images of the sensor’s surface recorded with a commercially available color camera. The different color channels (red and blue) were separated, analyzed digitally and divided by each other. Both measurement approaches show the same sensor characteristics but differentiate in terms of signal resolution and sensitivity due to different spectral sensitivities of the channels or filters. This shows that this sensor not only can be used for batch measurements but also for imaging and spatial resolution applications, as it has been proven to be useful in marine research by mapping oxygen concentration and pH distributions via imaging techniques [21].

## Experimental

### Materials

1H,5H,11H-[l]benzopyrano[6,7,8,ij]quinolizin-11-one (Coumarin 545T or C545T) and 3-(2-N-Methylbenzimidazolyl)-7-N,N-diethylaminocoumarin (Coumarin 30 or C30) were purchased from Sigma Aldrich Chemie GmbH (Steinheim, Germany). 3-(benzothiazol-2-yl)-7-(diethylamino)-2-oxo-2H-1-benzopyran-4-carbonitrile (Macrolex Fluorecent Red G or MFR) was purchased from Simon & Werner GmbH (Flörsheim am Main, Germany). Bromophenole blue (BPB), Tetraethoxysilane (TEOS) and celluloseacetate (CA) were obtained from Acros Organics (Geel, Belgium). Tetrahydrofuran (THF), N-Cyclohexyl-2-aminoethanesulfonic acid (CHES), acetic acid, sodium dihydrogen phosphate, disodium hydrogen phosphate and sodium chloride were bought from Carl Roth GmbH (Karlruhe, Germany). Hydrogel D1, D4 and D7 were obtained from CardioTech International Inc. (Wilmington, MA, United States). Millipore FHUP Fluoropore™ Membrane Filter was bought from EMD Millipore Corporation (Billerica, MA, United States). (Ru[dpp(SO_3_Na)_2__3_)Cl_2_) was synthesized in lab following to published results [40].

### Synthesis of reference particles

The reference particles were prepared analogically as reported before [41]. 3.86 g of TEOS was poured into a solution of 4 ml acetic acid, 1.25 ml and 0,02 mg Ru(dpp(SO_3_)_2_). The mixture was stirred vigorously right away for 5 minutes and after waiting 30 minutes it was filtered with a Macherey-Nagel MN 619 cellulose filter. The obtained particles were washed two times each with deionized water, ethanol, and acetone. In the end the particle were dried for 24 hours at 80°C in a drying oven.

### Sensor preparation

**Figure 7:**
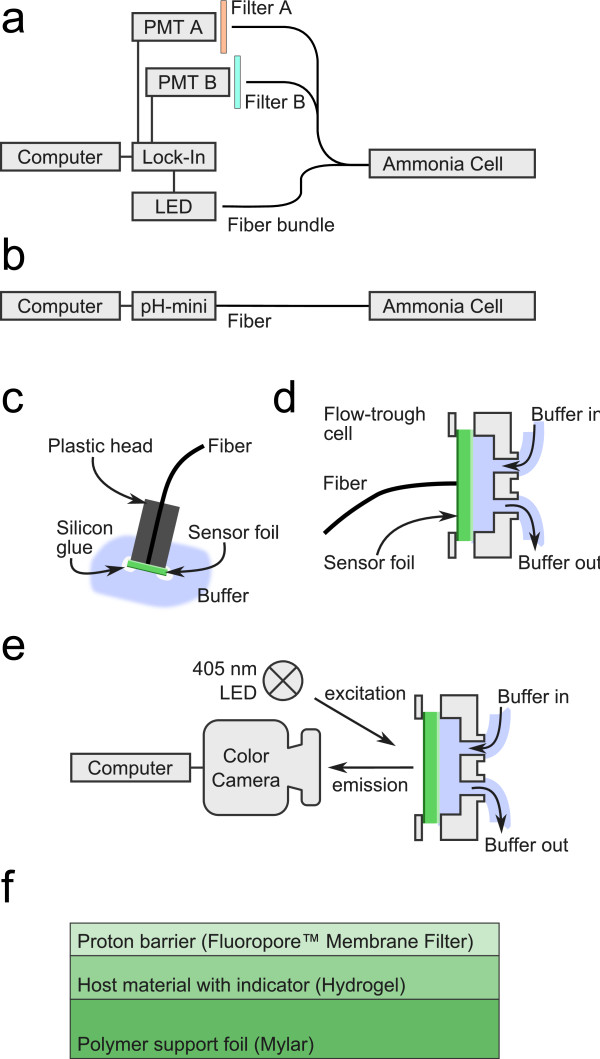
**Schemes of measurement setups, instrumentation setups and sensor cross section.** Homemade two filter read-out approach (**a**) for ratiometric measurements and pH-mini setup (**b**) for intensity based measurements. Plastic head sensor setup (**c**) for longtime measurements of multiple sensor spots, homemade flow-through cell (**d**) for all intensity based measurements and imaging setup (**e**) for chemical imaging. Schematic cross-section of the sensor foil (**f**). Sizes are not to scale.

The sensor layers were prepared by coating sensor cocktails onto a Melinex boPET foil (DuPont Teijin Films, Middlesbourgh, UK). Cocktails consisted of the two fluorescent dyes, the indicator, the polymer, and (in case of cocktails for DLR-referenced foils) the particles, dissolved in THF (see Table 1 for sensor cocktail composition). The cocktails were spread onto the foil using a drawdown bar film applicator (wet film thickness 25.4 μm) purchased from BYK-Gardner GmbH (Geretsried, Germany). Immediately after the spreading step, a FHUP Fluoropore™ membrane filter was laid onto the still wet sensor and pressed down with a brush. The THF evaporates through the filter, leaving a thin hydrogel layer between the membrane filter and the boPET foil (see Figure 7f for sensor cross section).

**Table 1:** Composition of different sensor cocktails (5%), all dissolved in THF (95%) (RP: reference particles)

**Sensor**	**Host material**	**FRET-System [% (v/v)]**	**Indicator [mmol/kg polymer]**
A	CA	C545T & MFR [2 / 0.25]	BPB [6]
B	D7	C545T & MFR [2 / 0.25]	BPB [6]
C	D4	C545T & MFR [2 / 0.25]	BPB [6]
D	D1	C545T & MFR [2 / 0.25]	BPB [6]
E	D7 & RP [80:20]	C545T & MFR [0.4 / 0.2]	BPB [6]
F	D7	C30 & MFR [2 / 0.2]	BPB [20]

### Buffer preparation

100 mM phosphate buffers (pH 7.2) containing different ammonia concentrations were prepared by dissolving sodium dihydrogen phosphate and disodium hydrogen phosphate in water [42]. An equivalent amount of ammonium chloride was dissolved in each buffer, resulting in a free ammonia (pK_a_ 9.25) concentration calculated by the Henderson-Hasselbach equation (see equation 2).

(2) $$pH=pK_{a}+\log_{10}\frac{c\left[NH_{3}\right]}{NH_{4}^{+}}$$

The temperature dependency of the ammonia-ammonium equilibrium was calculated by the Gibbs free energy (see equation 3)

(3) ΔG=R⋅T⋅1nK

The mathematical compensation of pK_a_ of ammonia towards salinity was based on a recently published study by Bell et al [36].

### Instrumentation and measurement

Absorbance spectra were recorded with a Varian Cary 50 UV-VIS spectrophotometer. Luminescence spectra were recorded using a Hitachi F-7000 fluorescence spectrometer. Intensity based and DLR-referenced sensor measurements were carried out with a pH-1 mini (PreSens, Germany). Two wavelength ratiometric measurements were carried out on a SR830 lock-in amplifier from Stanford Research Systems, Inc. (Sunnyvale, CA, United States), a 405 nm LED from Roithner Lasertechnik (Vienna, Austria) combined with two Hamamatsu photomultiplier tubes and two band pass filters (Carl Zeiss 575-625 (575 nm to 625 nm) and Horiba Scientific XF 1072 (460 nm to 490 nm)). Fluorescence imaging was carried out with an F-201C camera from Allied Vision Technologies (Stadtroda, Deutschland). A homemade flow-through cell was used for all intensity based response and calibration measurements calibration measurements. A series of plastic heads (to be plugged on the optical fibers of each readout system) with glued on sensor spots were used for longtime batch measurements. Figure 7 gives an overview over all sensor and instrumentation setups.

## Conclusion

An optical ammonia sensor was developed from commercially available products with simple means of manufacturing. The sensor exhibits a high sensitivity down to almost 1 μg/l ammonia, a response quicker than 120 seconds and virtually no cross sensitivity towards pH, temperature and salinity. Two different referencing methods have been presented to demonstrate a simple usability and possible application to imaging technologies.

## Abbreviations

BPB: Bromophenol blue; C30: Coumarin 30; C545T: Coumarin 545T; CA: Cellulose acetate; DLR: Dual lifetime referencing; FRET: Förster resonance energy transfer; MFR: Macrolex Fluorescent Red G; TWR: Two wavelength ratiometric.

## Competing interests

The authors declare that they have no competing interests.

## Authors’ contributions

TA planned and carried out the sensor manufacturing, measurements and data evaluation. BU assisted during imaging measurements and data evaluation of said measurements. IK provided scientific advices. TM headed the scientific planning and evaluation of the project. All authors have read and approved the final version.

## Authors’ information

All authors are working at the Institute of Analytical Chemistry and Food Chemistry, Graz University of Technology, Stremayrgasse 9, 8010 Graz, Austria.
